# Optimized PCR with sequence specific primers (PCR-SSP) for fast and efficient determination of Interleukin-6 Promoter ^-597/-572/-174^Haplotypes

**DOI:** 10.1186/1756-0500-2-245

**Published:** 2009-12-10

**Authors:** Michael Müller-Steinhardt, Friederike Schulte, Harald Klüter, Peter Bugert

**Affiliations:** 1 Institute of Transfusion Medicine and Immunology, Medical Faculty Mannheim, Heidelberg University, Friedrich-Ebert-Straße 107, 68167 Mannheim, Germany, DRK-Blutspendedienst Baden-Württemberg - Hessen, Germany

## Abstract

**Background:**

Interleukin-6 (IL-6) promoter polymorphisms at positions -597(G→A), -572(G→C) and -174(G→C) were shown to have a clinical impact on different major diseases. At present PCR-SSP protocols for IL-6 ^-597/-572/-174^haplotyping are elaborate and require large amounts of genomic DNA.

**Findings:**

We describe an improved typing technique requiring a decreased number of PCR-reactions and a reduced PCR-runtime due to optimized PCR-conditions.

**Conclusion:**

This enables a fast and efficient determination of IL-6 ^-597/-572/-174^haplotypes in clinical diagnosis and further evaluation of IL-6 promoter polymorphisms in larger patient cohorts.

## Findings

Interleukin-6 (IL-6) is a pleiotropic cytokine with a broad range of effects that is produced by a variety of different cells and plays a crucial role at the interface of adoptive and innate immunity. The increased knowledge about individual genetic susceptibility of the immune system led to the identification of three single biallelic nucleotide polymorphisms (SNP) within the promoter region of the IL-6 gene at positions -597(G→A) (rs1800797), -572(G→C) (rs1800796) and -174(G→C) (rs1800795) [1,2]. The three SNPs were shown to be in linkage disequilibrium and naturally occurring IL-6 ^-597/-572/-174^haplotypes have been characterized [2].

A number of recent studies investigated the clinical impact of the IL-6 promoter polymorphisms on different major diseases such as chronic obstructive pulmonary disease [3,4], viral infections [5], gout [6], osteoporosis [7], diabetes [8] or allograft survival [9]. Our own group recently presented evidence that the IL-6 promoter ^-597/-572/-174^genotype affects IL-6 secretion [10].

At present, typing of IL-6 ^-597/-572/-174^haplotypes requires an elaborate and time-consuming protocol [2]. A twelve-reaction PCR-SSP system with eight different allele-specific primers (AS1-AS8) is needed in order to identify both ^-597/-572/-174^haplotypes for the three biallelic sites in each individual tested. Since PCR-SSP is DNA-consuming, this procedure requires at least 500 ng genomic DNA; this, however, may be critical with regard to a retrospective analysis, or when valuable samples need to be analyzed. However for subsequent confirmatory investigations of larger patient cohorts a rapid and accurate genotyping technique is needed.

Here we report a modified PCR-SSP protocol which is suitable for the genotyping of IL-6 ^-597/-572/-174^haplotypes in the Caucasian population. It is faster, less labour-intensive and requires less DNA, since only four instead of twelve PCR reactions are necessary in order to detect all relevant ^-597/-572/-174^haplotypes in the Caucasian population (Table 1).

**Table 1:** Interleukin-6 Promoter ^-597/-572/-174^Haplotype Frequencies in different caucasian cohorts

*Haplotypes *major (minor)	*healthy blood donors (n = 100)* [Müller-Steinhardt et al. 2006]		*healthy controls (n = 182)* [Terry et al. 2000]		*kidney recipients (n = 158)* [Müller-Steinhardt et al. 2004]		*All (n = 440)*	
	**number**	**[%]**	**number**	**[%]**	**number**	**[%]**	**number**	**[%]**

GGG	113	56.5	195	53.6	165	52.2	473	53.8

AGC	74	37.0	147	40.4	135	42.7	356	40.4

GCG	11	5.5	19	5.2	15	4.7	45	5.1

GGC	2	1	2	0.6	1	0.3	5	0.6

(AGG)	0	0	1	0.3	0	0	1	0.1

(ACG)	0	0	0	0	0	0	0	0

(ACC)	0	0	0	0	0	0	0	0

(GCC)	0	0	0	0	0	0	0	0

total	200	100	364	100	316	100	880	100

For the isolation of genomic DNA from 200 μL whole blood, we used the QIAamp^® ^DNA Blood Mini Kit (Qiagen, Hilden, Germany) according to the manufacturer's standard protocol. The DNA concentration was estimated from the absorbance at 260 nm using a UV-spectrophotometer (GeneQuant *pro*; Amersham Biosciences, Freiburg, Germany). For validation, DNA samples (n = 100) from a previous study [10] genotyped according to the protocol by Terry et al. [2] were used.

Four forward (AS1-F1g, AS2-F3a, AS3-F3g, AS4-F3c) and two reverse primers (AS7-R1c, AS8-R1g) specific for the typing of the AGC (Primermix I), GGC (II), GGG (III) and GCG (IV) IL-6 ^-597/-572/-174^haplotype respectively were selected. While primers AS1-F1g, AS7-R1c and AS8-R1g have been adopted from the original protocol [2], primers AS2-F3a, AS3-F3g and AS4-F3c have been optimized for uniform PCR-conditions (Table 2).

**Table 2:** Primer Sequences and Combinations used in PCR-SSP-typing for Interleukin-6 Promoter ^-597/-572/-174^Haplotype

	Primermix	Primer Sequence	Product Size [bp]	- **597** Genotype	- **527** Genotype	- **174** Genotype	Detected major & (minor) Haplotypes
**I**	AS2-F3a AS8-R1g	5'-tgaagtaactgcacgaaatttgagga-3' 5'-tgcaatgtgacgtcctttagcatg-3'	473	A	-	C	AGC (ACC)

**II**	AS1-F1g AS8-R1g	5'-aagtaactgcacgaaatttgaggg-3' 5'-tgcaatgtgacgtcctttagcatg-3'	471	G	-	C	GGC (GCC)

**III**	AS3-F3g AS7-R1c	5'-grtggccaggcagttctacaacagccg-3' 5'-tgcaatgtgacgtcctttagcatc-3'	449	-	G	G	GGG (AGG)

**IV**	AS4-F3c AS7-R1c	5'-grtggccaggcagttctacaacagccc-3' 5'-tgcaatgtgacgtcctttagcatc-3'	449	-	C	G	GCG (ACG)

	β-globF4 β-globR5	5'-gcttaccaagctgtgattcc-3' 5'-aaggtgcccttgaggttgtc-3'	731				

According to an optimized protocol for PCR-SSP [11] reactions were carried out in a total volume of 10 μL, containing 20 ng DNA, 1 μM each of the various allele-specific forward and reverse primers, 0.2 μM each of the internal control primers, 10 mM Tris-HCl, 50 mM KCl, 1.5 mM MgCl2, 0.01% BSA, 5% glycerol, 0.1 mg/mL cresol red, and 0.4 U *Taq *DNA polymerase.

PCR-SSP was performed with the following cycling program: a 2-min initial denaturation at 95°C, followed by 10 cycles of 15 s denaturation at 95°C and 1 min annealing/extension at 65°C, followed by 20 cycles of 15 s denaturation at 95°C, 1 min annealing at 61°C, and 30 s extension at 72°C. Using a PTC-200 cycler (BioRad), the total runtime of the PCR-program was 68 min. The amplification products were separated on 2% agarose gels containing 0.5 ng/mL ethidium bromide in a rapid agarose gel electrophoresis (RAGE^®^; Cascade Biologics, Portland, OR, USA) chamber for 5 min at 25 V/cm. The results were obtained by a visual inspection of the gels and documented using a UV documentation device with charge-coupled device (CCD) camera (UVP, Upland, CA, USA).

**Figure 1:**
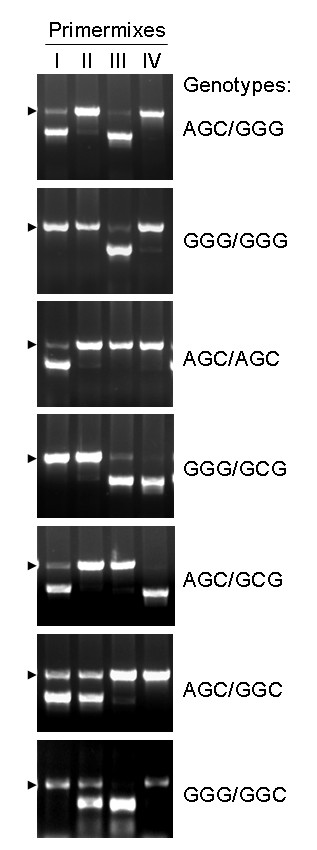
**Validation of the optimized PCR-SSP protocol by typing of seven reference DNA-samples**. Two primers specific for the typing of the AGC (Primermix I), GGC (II), GGG (III) and GCG (IV) IL-6 ^-597/-572/-174^haplotype respectively were used. The amplification product (731 bp) of the internal control is marked by an arrowhead. Genotyping results were as expected (AGC/GGG, GGG/GGG, AGC/AGC, GGG/GCG, AGC/GCG, AGC/GGC, GGG/GGC).

First-line validation was carried out by genotyping seven reference samples representing all relevant ^-597/-572/-174^haplotypes of the Caucasian population (GGG, AGC, GCG, GGC). The genotypes were as follows: AGC/GGG, GGG/GGG, AGC/AGC, GGG/GCG, AGC/GCG, AGC/GGC and GGG/GGC. Our modified PCR-SSP technique confirmed the typing results of the reference samples in all cases (Figure 1).

Additional validation of the technique could be achieved by retyping 100 healthy blood donor samples previously genotyped according to the protocol by Terry et al. [10]. Our modified technique confirmed all previous typing results unambiguously (data not shown).

PCR-SSP techniques are widely employed for the genotyping of SNPs. After PCR and agarose gel electrophoresis, the genotyping result is evaluated by the presence or absence of an allele-specific PCR product. Moreover, PCR-SSP-typing is suitable for haplotyping of neighbouring SNPs; furthermore, the presence of two alleles on one chromosome can be demonstrated when two appropriate allele-specific primers are combined in a single PCR-reaction. Consequently, complete typing of three neighbouring biallelic SNPs requires twelve PCR-reactions! Since this procedure is very extensive and time-consuming it is not suitable for the typing of larger cohorts.

In our study, we describe a modified PCR-SSP protocol which is suitable for rapid IL-6 ^-597/-572/-174^haplotyping focusing on the four major ^-597/-572/-174^haplotypes (AGC, GGC, GGG, GCG) representing 99.9% of all haplotypes in the Caucasian population (Table 1). Since only four, instead of twelve, PCR-reactions are needed and a protocol with optimized sensitivity is used [11], the amount of required genomic DNA could be reduced from 500 to 100 ng per genotype. This can be extremely valuable, especially if samples of limited quantity have to be analyzed.

Notably, our protocol does not distinguish between the GGG and AGG ^-597/-572/-174^haplotype, which has only been detected in one Caucasian individual so far (Table 1). However, as its frequency is extremely low (0.1%), this would not affect the overall outcome of association studies. Furthermore, ACC, ACG and GCC also represent minor ^597/-572/-174^haplotypes that cannot be distinguished likewise. Even though they have not been observed among Caucasians and are only of theoretical importance, it cannot be ruled out that they might occur in other populations as well.

In order to run all four PCR-reactions under identical conditions, we optimized the selection of the allele-specific primers. Thereby, the runtime of the PCR-procedure could be reduced from 110 to 68 min with an overall analysis time of approximately 2 hours including all pipetting procedures, DNA-isolation (30 min), electrophoresis (10 min) and documentation (5 min).

In summary, we optimized a previous PCR-SSP protocol for IL-6 ^-597/-572/174^haplo-typing of Caucasian individuals with regard to the number of PCR-reactions, the amount of genomic DNA required and overall runtime. This method represents an important prerequisite for further evaluating the clinical impact of IL-6 promoter polymorphisms in larger cohorts.

## Competing interests

The authors declare that they have no competing interests.

## Authors' contributions

MMS conceived the study, interpreted results and drafted the manuscript, FS performed SNP-typing and assisted in data analysis, HK participated in study design and provided valuable comments, PB conceived the study, designed the primers and interpreted results. All authors read and approved the final manuscript.
